# LOST to follow-up Information in Trials (LOST-IT): a protocol on the potential impact

**DOI:** 10.1186/1745-6215-10-40

**Published:** 2009-06-11

**Authors:** Elie A Akl, Matthias Briel, John J You, Francois Lamontagne, Azim Gangji, Tali Cukierman-Yaffe, Mohamad Alshurafa, Xin Sun, Kara A Nerenberg, Bradley C Johnston, Claudio Vera, Edward J Mills, Dirk Bassler, Arturo Salazar, Neera Bhatnagar, Jason W Busse, Zara Khalid, SD Walter, Deborah J Cook, Holger J Schünemann, Douglas G Altman, Gordon H Guyatt

**Affiliations:** 1 Departments of Medicine and Family Medicine, State University of New York at Buffalo, Buffalo, USA; 2 Department of Biostatistics and Clinical Epidemiology, McMaster University, Hamilton, Canada; 3 Basel Institute for Clinical Epidemiology, University Hospital Basel, Basel, Switzerland; 4 Department of Medicine, McMaster University, Hamilton, Canada; 5 Department of Medicine, University of Sherbrooke, Sherbrooke, Canada; 6 Gertner Institute for Epidemiology & Health Policy Research, Sheba medical center, Sackler school of Medicine, Tel -Aviv University, Tel-Aviv, Israel; 7 Department of Clinical Epidemiology, West China Hospital, Sichuan University, Chengdu, PR China; 8 Department of Medicine, University of Alberta, Edmonton, Canada; 9 Department of Obstetrics & Gynecology, Pontificia Universidad Católica de Chile, Santiago, Chile; 10 Centre for International Health and Human Rights Studies, North York, Canada; 11 Department of Neonatology, University Children's Hospital, Tuebingen, Germany; 12 Department of Internal Medicine, Wayne State University, Detroit, USA; 13 Health Sciences Library, McMaster University, Hamilton, Canada; 14 The Institute for Work & Health, Toronto, Ontario, Canada; 15 Department of Epidemiology, Italian National Cancer Institute Regina Elena, Rome, Italy; 16 Centre for Statistics in Medicine, University of Oxford, Oxford, UK

## Abstract

**Background:**

Incomplete ascertainment of outcomes in randomized controlled trials (RCTs) is likely to bias final study results if reasons for unavailability of patient data are associated with the outcome of interest. The primary objective of this study is to assess the potential impact of loss to follow-up on the estimates of treatment effect. The secondary objectives are to describe, for published RCTs, (1) the reporting of loss to follow-up information, (2) the analytic methods used for handling loss to follow-up information, and (3) the extent of reported loss to follow-up.

**Methods:**

We will conduct a systematic review of reports of RCTs recently published in five top general medical journals. Eligible RCTs will demonstrate statistically significant effect estimates with respect to primary outcomes that are patient-important and expressed as binary data. Teams of 2 reviewers will independently determine eligibility and extract relevant information from each eligible trial using standardized, pre-piloted forms. To assess the potential impact of loss to follow-up on the estimates of treatment effect we will, for varying assumptions about the outcomes of participants lost to follow-up (LTFU), calculate (1) the percentage of RCTs that lose statistical significance and (2) the mean change in effect estimate across RCTs. The different assumptions we will test are the following: (1) none of the LTFU participants had the event; (2) all LTFU participants had the event; (3) all LTFU participants in the treatment group had the event; none of those in the control group had it (worst case scenario); (4) the event incidence among LTFU participants (relative to observed participants) increased, with a higher relative increase in the intervention group; and (5) the event incidence among LTFU participants (relative to observed participants) increased in the intervention group and decreased in the control group.

**Discussion:**

We aim to make our objectives and methods transparent. The results of this study may have important implications for both clinical trialists and users of the medical literature.

## Background

In this paper, we define loss to follow-up as incomplete ascertainment of the primary outcome for participants randomized in the trial. If the authors exclude participants from the analysis, but still provide their primary outcome data (thus allowing others to conduct an analysis consistent with the intention-to-treat principle), we will consider that loss to follow-up did not occur. However, if the authors do not provide the primary outcome data of those excluded participants, we will consider that loss to follow-up did occur.

In randomized controlled trials (RCTs) assessing health care interventions, adherence to key methodological principles helps ensure the validity of the results. The intent of randomization is to ensure that the treatment and control groups are similar at baseline with respect to both known and unknown prognostic factors and confounders. Blinding the trial participants and study personnel to treatment allocation is intended to ensure that the care provided (aside from the experimental intervention) and the methods for outcome assessment are similar in between groups. Ensuring that all randomized patients are analyzed according to the group to which they were randomized preserves the prognostic balance created by randomization and maintained by blinding.

Incomplete ascertainment of patient outcomes for the final analysis due to loss to follow-up may bias the results if the reasons for unavailability of participant data are associated with the outcome of interest. Attributing outcomes to patients according to the groups to which they were randomized cannot protect from bias if one doesn't know what those outcomes were [1]. Investigators can reduce the amount of missing data, but in most instances cannot eliminate it [2]. How to best deal with missing data remains controversial; one interpretation of the "intention to treat principle" is that it requires imputing the missing data [3]. The Consolidated Standards of Reporting Trials (CONSORT) flow diagram is intended to render explicit the information necessary to make a judgment about loss to follow-up and attribution of events to patients according to randomization (or otherwise) [4,5].

The extent of loss to follow-up in RCTs and the investigators' approach to dealing with the problem has been the subject of a number of studies. Hollis et al. surveyed all RCTs published in 1997 in 4 "top" medical journals (n = 249) and found that 75% of the trials had missing data for the primary outcome. The most frequently used strategy (45%) for handling missing data was to restrict analysis to those with full outcome information (complete case analysis) [6].

Wood et al. examined all RCTs published over 6 months in 2001 in 4 "top" medical journals (n = 71) and found that 89% of trials had missing outcome data, 65% of which used complete case analysis in their primary analysis [7]. In a more recent study of RCTs reported in 10 medical journals in 2002, Gravel et al. found that more than 60% of articles claiming the use of an intention-to-treat approach (n = 249) had missing data in their primary analysis with few articles reporting a specific strategy for dealing with missing data [8].

Baron et al. examined the rate of missing data in superiority trials published between 1994 and 2003 that assessed structural outcomes in rheumatic diseases (n = 81) [9]. They were able to determine the rate of missing data in 78% of reports; in approximately one-third of these reports the rate was >20%. Only 24% reported statistical methods for handling missing data. Although missing data is not as problematic in interpreting the results of an individual trial with statistically non-significant effect estimates, none of the three aforementioned studies distinguished between negative and positive studies when reporting about handling missing data.

Several studies have evaluated the association between the extent of reported loss to follow-up and the magnitude of treatment effects. Among 190 primary RCTs from 14 meta-analyses (search year 2000), Kjaergard et al. found that reported follow-up was not significantly associated with estimated intervention effects [10]. In a study of 276 RCTs from 26 meta-analyses (search year 2000), Balk et al. found that recording of patients lost to follow-up (LTFU), the reason for dropouts, and percentage of enrolled patients who dropped out, were not statistically significantly associated with treatment effect [11]. In a 1995 study of 250 controlled trials from 33 meta-analyses, Schulz et al. found that excluding participants after randomization was not significantly associated with the magnitude of effect [12]. Tierney et al. analyzed 14 meta-analyses of individual patient data to investigate whether patients' exclusion from analysis affected the results of trials and meta-analyses [13]. Considering individual trials, they did not find evidence that exclusion of patients altered their results. However, meta-analyses based only on 'included' patients tended to favor the intervention under investigation.

Thus, in spite of the theoretical concerns, no study has found evidence of a statistically significant association between loss to follow-up and the magnitude of treatment effect. There are a number of possible explanations for this apparent lack of association. The first possibility is that loss to follow-up does not bias trial results. However, this explanation is unlikely in the light of evidence that patients LTFU often have worse outcomes than those who are not LTFU [14,15]. The second possibility is that the association between loss to follow-up and treatment effect is confounded by the adequacy of reporting loss to follow-up. Schulz et al showed that trials reporting exclusions were generally of a higher methodological quality than those that did not. This suggests that exclusions may have been present but not reported in some trials of lower methodological quality [16]. A third possibility is that loss to follow-up does bias the treatment effect but the rates of loss to follow-up are low enough that the above mentioned studies lacked power to detect any association. A final possibility is that loss to follow-up leads to bias that varies in direction both between trials leading to an overall lack of association when considering a number of trials together.

The primary objective of this study is to assess the potential impact of loss to follow-up on the estimates of treatment effect. The secondary objectives are to describe, for published RCTs, (1) the reporting of loss to follow-up information, (2) the analytic methods used for handling loss to follow-up information, and (3) the extent of reported loss to follow-up.

## Methods

### Overall study design

We will conduct a systematic review of reports of RCTs recently published in five general medical journals with the highest citation rates. Eligible trials will report statistically significant effect estimates with respect to primary outcomes that are patient-important and expressed as binary data.

As mentioned earlier, we define loss to follow-up as incomplete ascertainment of the primary outcome for subjects randomized in the trial. If the authors exclude participants from the analysis, but still provide their primary outcome data (thus allowing others to conduct an analysis consistent with the intention-to-treat principle), we will consider that loss to follow-up did not occur. However, if the authors do not provide the primary outcome data of those excluded participants, we will consider that loss to follow-up did occur.

### Eligibility criteria

The inclusion criteria are:

1. Study published in the five general medical journals ranked the highest according to the number of citations reported in the Journal Citation Report (JCR) by Thomson ISI (Institute for Scientific Information) in 2008: New England Journal of Medicine (NEJM), Lancet, Journal of the American Medical Association (JAMA), British Medical Journal (BMJ), and Annals of Internal Medicine.

2. Study is described by its authors as a RCT;

3. Primary outcome is a patient-important outcome (defined below);

4. Binary data, allowing the construction of a 2 × 2 table, are reported for the primary outcome. This includes binary summaries of continuous outcomes (e.g. pain reduction of 50% or more), or of time to event outcomes (e.g. proportion surviving to 2 years). Trials with time to event outcomes qualify only if data for 2 × 2 tables are available and an effect estimate (i.e. relative risk) calculated from the 2 × 2 table remains statistically significant. We acknowledge that the proportion we are calculating for time to event data (number of subjects with events divided by number randomized) as well as the upper and lower values of its confidence interval are not exactly risks and plan a sensitivity analysis excluding these trials.

The exclusion criteria are:

1. Study described as RCT but is not truly randomized. We will use Cochrane criteria for judging risk of bias [17] as related to sequence generation to judge whether a study is truly a RCT;

2. Cluster RCT;

3. Cross-over RCT;

4. n-of-1 RCT;

5. RCT reported in a research letter

6. Meta-analysis of 2 or more previously published RCTs;

7. Primary outcome is not statistically significant. (See Appendix 1 for LOST-IT rules for judging statistical significance);

8. Primary outcome is a composite endpoint with at least one component not patient-important;

9. Primary outcome based on registries (e.g. death registry), if the authors do not report any other eligible outcome;

10. Primary outcome is binary but is reported only as a rate (e.g. 100 death/10,000 person years).

The unit of analysis for the LOST-IT study will be a report of an RCT, not the RCT itself. Consequently, we may include more than one report of the same RCT as long as each meets the eligibility criteria. For example, we will include, if otherwise eligible, reports of secondary analyses, reports of subgroup analyses, and reports of longer follow-up of RCTs, even if the allocation code was broken.

The rationale for the decision to include reports of secondary and sub-group analyses is that, conceptually, we are interested in the extent to which any reports of RCTs in a set of prestigious journals may be misleading as a result of LFUP.

For multiple-arm RCTs and RCTs with a factorial design, we will only consider a comparison with statistically significant results. If there is more than one comparison with statistically significant results, we will choose the one with the largest effect estimate expressed in relative terms (e.g., relative risk reduction). For RCTs with multiple follow-up times, we will use the longest follow-up time that is associated with a statistically significant result.

### Patient-important outcomes

Patients typically assign varying importance to different health outcomes [18]. We define a patient-important outcome as an outcome for which one would answer with "yes" the following question: "if the patient knew that this outcome was the only thing to change with treatment, would patient consider receiving this treatment if associated with side effects or cost?" To focus our study on patient-important outcomes and to explore our findings across different levels of patient importance, we developed a hierarchy of outcomes (Appendix 2). This hierarchy is adapted from that used in another study of cardiovascular outcomes [19] and was developed through consensus discussions of the relative importance of outcomes identified from RCTs eligible for inclusion in the current study. Categories I, II, and III but not category IV include patient-important outcomes.

### Categories of loss to follow-up

We will use the following mutually exclusive categories of subjects to classify reasons for potential loss to follow-up:

1. Mistakenly randomized with inappropriate post-randomization exclusion;

2. Not received intervention with inappropriate post-randomization exclusion;

3. Withdrew consent;

4. Non-adherent;

5. Crossed-over;

6. Lost contact.

**Figure 1:**
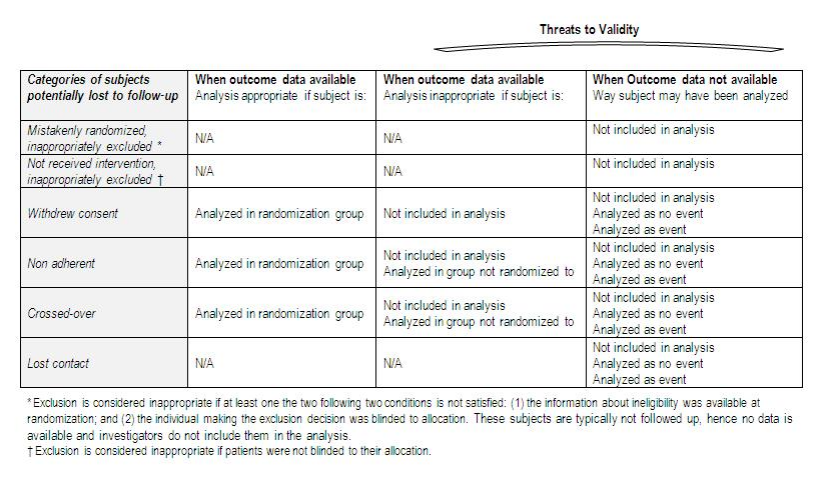
**LOST-IT framework illustrating issues related to loss-to-follow-up and threats to validity for binary outcomes**.

Mistakenly randomized subjects are those that were ineligible at the time of randomization. We will consider post-randomization exclusion inappropriate if either: (1) the information about ineligibility was not available at randomization; or (2) the individual making the exclusion decision was not blinded to allocation. For participants who did not receive the intervention we will consider their post-randomization exclusion inappropriate if patients were not blinded to their allocation. For survival data, we will not consider as LTFU those subjects that are censored because of the planned termination of study. We will consider all post randomization exclusions related to study center exclusion as appropriate. Figure 1 shows these different categories within the LOST-IT framework illustrating issues related to loss to follow-up and threats to validity for binary outcomes.

### Literature search

We will electronically search Medline (OVID interface) using the Cochrane Collaboration's "highly sensitive" search strategy to identify reports of RCTs in Medline (Additional file 1). The search will be restricted to human studies.

### Review process

Reviewers trained in health research methodology will work in pairs. They will perform each of the review stages (selection of studies, selection of the primary outcome, and data abstraction) in duplicate and independently. They will attempt to resolve any disagreements by discussion and, when unsuccessful, with the assistance of an arbitrator. One person (EAA) will serve as the arbitrator for all studies. For resolving disagreements related to data abstraction, the arbitrator will independently abstract data prior to discussions with the 2 reviewers. Before starting data abstraction, we will conduct calibration exercises to ensure consistency between reviewers, through sampling papers from the 5 journals under review.

We will use paper forms for title and abstract screening and electronic forms, hosted by a university central server, for full text screening and data abstraction. The full text articles of potentially eligible RCTs (and supporting documentation) will also be available electronically on the central server.

### Selection of studies

Teams of 2 reviewers will screen the titles and abstracts of identified citations for potential eligibility using a standardized, pilot-tested, title and abstract screening form with corresponding detailed written instructions (Additional file 2). They will document the screening outcome and reason for exclusion (if applicable). The reviewers will then screen the full texts of potentially eligible RCTs for eligibility. They will use a standardized, pilot-tested full text screening form with corresponding detailed written instructions.

### Selection of the primary outcome

Before extracting data from an eligible RCT, teams of 2 reviewers will select the primary outcome as follows. If the report specifies a single primary outcome, we will select it as the primary outcome for LOST-IT. If the report specifies primary outcomes for both efficacy and safety, we will select the primary efficacy outcome. If the report specifies multiple primary outcomes, we will select the statistically significant outcome in the highest category on the outcome hierarchy (Appendix 2). If the report does not specify a primary outcome, we will select the outcome with the highest category on the outcome hierarchy that is statistically significant.

### Data abstraction

Reviewer teams will abstract data from eligible RCTs using a pilot-tested standardized data abstraction form (Additional file 3) with corresponding detailed instructions. We will also search for and use additional data related to the RCT of interest reported in ACP Journal Club, errata, or trial protocols published in peer review journals. For LTFU information, however, we will use only data from the report under consideration (e.g. we will not use for a report of a subgroup analysis a participant flow diagram from the original report not considering the specific subgroup).

We will extract information about the study background, methodological quality, reporting of loss to follow-up, analytic methods used for handling LTFU information, and loss to follow-up statistical data regarding the primary analysis of the primary outcome as selected for LOST-IT (See Additional file 4 for more details about reporting of loss to follow-up, analytic methods used for handling loss to follow-up information, and loss to follow-up statistical data).

We will determine the follow-up status and analysis of each category of subjects potentially lost to follow-up (Figure 1) as follows: (1) unclear whether followed up, (2) not followed up, (3) followed-up, not included in the analysis, (4) analyzed in a group to which they were not randomized (for crossed over and non-adherent). We will also determine for each category whether loss to follow-up was reported to be related to side effects or adverse events, or other specified reasons.

We will always consider the negative aspect of an outcome (e.g. mortality instead of survival). We will define participants' non-adherence to the intervention according to the author's definition. When there is no explicit statement (or flow diagram) indicating that no participants were LTFU, we will assume that this was actually the case if the following 3 conditions are met:

i. all protocol deviations were explicitly described and detailed;

ii. the authors explicitly stated that all enrolled participants were included in the analyses

iii. the outcome is mortality, as it is less likely that the investigators would have LTFU for that outcome.

We will contact the authors and send them the results of our data abstraction to give them the opportunity to confirm them or refute them.

### Sample size

We estimate that we will identify about 200 eligible papers by applying our search strategy to the years 2005–2007. A sample size of 200 would result in the following confidence intervals for 3 plausible proportions (10%, 20% and 30%) of articles losing statistical significance in our analysis: (5.84–14.16); (14.46–25.54); and (23.65–36.35) respectively. We find these confidence intervals to be acceptable.

### Analysis

#### Agreement

We will assess agreement between reviewers for study inclusion both at the title and abstract screening and the full text screening stage. We will calculate both crude agreement and chance-corrected agreement (kappa statistic). If fewer than 15% or more than 85% of citations are included in this study, we will measure agreement using chance-independent agreement (phi statistic). We will interpret the agreement statistics using the guidelines proposed by Landis and Koch [20]: kappa values of 0 to 0.20 represent slight agreement, 0.21 to 0.40 fair agreement, 0.41 to 0.60 moderate agreement, 0.61 to 0.80 substantial agreement, and greater than 0.80 almost perfect agreement. We will use these same thresholds for interpreting phi.

#### Reporting of loss to follow-up information

We will calculate the following (denominator = number of RCTs included in the review):

1. the proportion of RCTs that explicitly reported whether LTFU occurred or not (either in general or specifically relating to the above defined categories);

2. the proportion of RCTs that reported a CONSORT flow diagram with LTFU provided;

3. the proportion of RCTs that reported loss to follow-up separately for the 2 comparison groups;

4. the proportion of RCTs that reported loss to follow-up at each planned outcome assessment;

5. the proportion of RCTs that compared the baseline characteristics of participants LTFU to those not LTFU and the proportion that compared the baseline characteristics of those LTFU in intervention and control groups;

6. the proportion of trials that discussed the implications of loss to follow-up in the context of their findings

#### Analytic methods used for handling LTFU information

We will calculate the proportion of RCTs that reported the analytic methods used for handling LTFU information. We will also calculate among RCTs reporting loss to follow-up information:

1. the percentage of RCTs, using the term "intention to treat" and "modified intention to treat";

2. the percentages of RCTs with different types of post randomization exclusions (i.e. mistakenly randomized, no intervention received and center exclusion) that are respectively appropriate and inappropriate;

3. the percentage of RCTs in which participants for whom outcome data is available were analyzed in the group to which they were randomized.

4. The analytical method used for dealing with LTFU information in the primary analysis and any additional analyses of the primary outcome.

#### Extent of reported loss to follow-up

For each trial, we will calculate (for each group and for the entire cohort) the percentage of total participants classified as LTFU according to our study definition. We will then calculate the mean and standard deviation of the percentage across trials. We will repeat the calculations for the following 3 categories:

• *Not followed up*: mistakenly randomized and inappropriately excluded; not received intervention and inappropriately excluded; withdrew consent and not followed up; cross over and not followed up; non adherent and not followed up; lost contact with participants and no other source of outcome data;

• *Followed up but not included in the analysis*: withdrew consent, followed up but not included in the analysis; cross over, followed up but not included in the analysis; non-adherent, followed up but not included in the analysis;

• *Unclear whether followed up*: withdrew consent and unclear whether followed up; cross over and unclear whether followed up; non adherent and unclear whether followed up;

• *Analyzed in a group to which they were not randomized*: cross over, followed up but analyzed in a group to which they were not randomized; non-adherent, followed up but analyzed in a group to which they were not randomized.

For each trial, we will also calculate for each group and for the entire trial the "LTFU to events ratio" (i.e. the ratio of the total number of participants classified as LTFU, according to this study definition, to the number of primary outcome events). We will then calculate the mean and standard deviation of the ratio across trials.

We will then conduct regression analyses with "percentage of participants LTFU" as the dependent variable. We will use two sets of independent variables:

• General trial characteristics: number of study centers, type of funding, type of outcome (mortality vs. others), clinical area (medical vs. surgical), type of intervention (pharmacological vs. surgery/invasive procedure vs. other), and length of follow-up

• Methodological trial characteristics: concealing allocation, blinding, stopping early for benefit, reporting the use of "ITT" analysis, and analyzing participants as randomized.

We will re-categorize the variables funding, intervention, and blinding and consider them for the regression model on the basis of their empirical distribution. We will use weighted linear regression models, with the weight for each observation being defined as the inverse of the estimated variance of the observed outcome (dependent) variable. This weighting has the effect of giving more emphasis to observations that are relatively precise (and subject to less sampling variation), and corresponding less emphasis to observations with greater sampling variation. It also has the advantage of satisfying the regression assumption of homogeneity in the (weighted) residuals. We will include only studies reporting LTFU information.

#### Potential impact of loss to follow-up

We will conduct analyses to test the effect of different assumptions about the outcomes of participants LTFU on the effect estimate for the primary outcome. We will use data from the 2 × 2 tables for all these analyses. For papers reporting a survival analysis we will include the LTFU participants in the denominator. For each of the assumptions, we will calculate (1) the percentage of RCTs that lose statistical significance; and (2) the mean change in effect estimate across RCTs. We will compare the relative risk (RR) for each assumption with the RR based on the 2 following "base cases":

1. A complete case analysis excluding LTFU participants from both the denominator and numerator;

2. The approach to generating effect estimate reported by the investigators.

While including LTFU participants in the denominator we will test the following assumptions about the events among participants LTFU:

1. None of the LTFU participants had the event;

2. All LTFU participants had the event;

3. All LTFU participants in the treatment group had the event; none of those in the control group had it (worst case scenario);

4. LTFU participants had higher event incidences than the observed participants in their respective randomization groups, but the relative increase was higher for the intervention group (e.g. 30% for the intervention group, 10% for the control group). We define RI_LTFU/FU _as the relative increase in event rate of those LTFU compared to those followed up. We will pre-specify a number of combinations of RI_LTFU/FU _for the intervention and control groups. The maximum possible value of RI_LTFU/FU _for a specific event rate corresponds to an increase of that event rate to 100%. Tables 1 and 2 in Additional file 5 represent the dummy tables for these analyses and the values of RI_LTFU/FU _are just illustrative. We continue to explore the literature for evidence to support our choices;

5. LTFU participants in the intervention group had a higher event incidence than their randomization group, and LTFU participants in the control group had a lower event incidence than their randomization group. We define RD_LTFU/FU _as the relative decrease in event rate of those LTFU compared to those followed up. We will pre-specify a number of combinations of RI_LTFU/FU _for the intervention and RD_LTFU/FU _for the control group. Tables 3 and 4 in Additional file 5 represent the dummy tables for these analyses.

We will then conduct regression analyses with "delta log of effect estimate" as the dependent variable. We will use two sets of independent variables:

• General trial characteristics: number of study centers, type of funding, type of outcome (mortality vs. others), clinical area (medical vs. surgical), type of intervention (pharmacological vs. surgery/invasive procedure vs. other), and length of follow-up

• Methodological trial characteristics: concealing allocation, blinding, stopping early for benefit, reporting the use of "ITT" analysis, and analyzing participants as randomized.

We will re-categorize the variables funding, intervention, and blinding and consider them for the regression model on the basis of their empirical distribution. We will use weighted linear regression models, with the weight for each observation being defined as the inverse of the estimated variance of the observed outcome (dependent) variable.

The primary analyses will include all studies while the sensitivity analyses will exclude studies reporting time-to-event data

## Discussion

This protocol describes a methodological study the goal of which is to assess the potential impact of loss to follow-up information in published RCTs on the estimates of treatment effect. By publishing our detailed study protocol we make our objectives and methods transparent [21].

Strengths of our study include its transparent and systematic methods with respect to searching for eligible reports, selecting eligible studies, selecting the primary outcomes and abstracting data. We will ensure data integrity by preparing detailed written instructions, conducting formal calibration exercises, and measuring agreement between pairs of reviewers.

We will focus on reports of trials with statistically significant effect estimates published in major general medical journals because these studies are more likely to influence clinical practice than studies with non-significant effect estimates or studies published in lower profile journals. In addition missing data is not as problematic in interpreting the results of an individual trial with non-statistically significant effect estimates. This decision is likely to affect the representativeness of the reports we are including and our ability to completely uncover the potential problems. There is indirect evidence that RCTs published in top medical journals tend to report larger effect estimates than those published in lower profile journals [22]. In addition, RCTs published in top medical journals are typically of higher methodological quality [23] and thus likely to suffer from lower rates of loss to follow-up. Given that the trial reports we are focusing on report larger effect estimates and lower rates of loss to follow-up, our findings will likely represent a "best case" scenario of the potential impact of loss to follow-up information on the estimates of treatment effect in published RCTs.

The type of outcome and the timing of its assessment are likely to be associated with the extent of loss to follow-up. Loss to follow-up is, for instance, likely to be lower for survival compared to a measurement of quality of life. Similarly, in a trial of an analgesic, loss to follow-up is likely to be lower for pain at day 2 compared to pain at 1 year. We are thus categorizing the type of outcome and recording the timing of its assessment. Our use of a newly developed outcome hierarchy for selecting and categorizing the primary outcome for each trial may be questionable. We have, however, used a systematic approach to adapt a previous hierarchy. Our approach in developing this previous hierarchy, moreover, was accepted by editors and reviewers of a prominent journal and has not, to our knowledge, elicited adverse criticism. We will use this hierarchy only for reports not specifying the primary outcome of the trial – the number of which should be relatively small. Finally, we are selecting the primary outcome in duplicate and independent fashion which will allow us to establish the reproducibility of the judgment.

We have specifically chosen not to include RCTs reporting primary outcomes that are continuous variables or expressed as rates, because of specific challenges related to analyzing and reporting LTFU information in those trials, e.g. use of last value carried forward with continuous outcomes [3]. We hope to conduct a separate study for continuous variables. For the same reasons, we did not include RCTs that have a cluster or cross-over design. Consequently, our results will be pertinent to parallel arm RCTs and factorial RCTs reporting results in a binary fashion.

To assess the potential impact of loss to follow-up information on effect estimates we will consider a number of assumptions about the outcomes of participants LTFU. The first 2 assumptions (none of the LTFU participants had the event, all LTFU participants had the event) are less plausible but frequently used and/or discussed in the literature. The 3^rd ^assumption (worst case scenario) is an extreme and in most cases unrealistic assumption. However, it is valuable in demonstrating the robustness of a trial results when the effect estimate remains statistically significant even under the assumption of a worst-case scenario. The remaining 2 assumptions (assuming that LTFU participants have different event incidences than the observed group) are more plausible and designed (in terms of differential extent and direction between intervention and control) to test the robustness of effect estimates. The challenge is to choose plausible values for RI_LTFU/FU _and RD_LTFU/FU_. The values we use in our dummy tables are just illustrative and we are continuing to explore the literature for evidence to support our choices. For example, a recent report on antiretroviral therapy scale-up programs in Africa found that the incidence of death among participants LTFU was 5 times as high as the incidence in those followed-up [14]. While other imputation methods such as regression models and multiple imputations might be preferable, they are not feasible because they would require raw data from each included study.

The results of this study should have important implications for trialists. Evidence of vulnerability will call for improving both trial design and implementation, to minimize LTFU. The results of our study will also have important implications for clinicians interpreting the findings of RCTs. Our findings will uncover the potential impact of plausible assumptions about the outcome of participants LTFU on the results of those positive studies that are most likely to affect clinical practice. They might provide reassurance that these results are usually – at least for reports in five prestigious journals – robust or, on the contrary, suggest that many high-profile trials are vulnerable. We may also find that results vary in robustness and users of the literature will have to evaluate them on a case by case basis using clinically plausible assumptions. Our fondest hope is to help create a culture in which investigators uniformly test alternative assumptions regarding LTFU and discuss the extent of the vulnerability of their findings to varying assumptions regarding LTFU.

## Competing interests

The authors declare that they have no competing interests.

## Authors' contributions

All authors contributed the design of this protocol.

## Appendix 1

### LOST-IT rules for judging statistical significance

The rules for judging statistical significance will be the following:

• p-value < 0.05;

• CI not including 1.0 when no p-value is reported;

• Statistical significance is relative to the null hypothesis that difference between groups is 0, irrespective of whether the study was a superiority, equivalence, or non-inferiority trial;

• If both intention to treat and per protocol analyses are reported, we will consider the statistical significance of the former;

• If both unadjusted and adjusted analyses are reported, we will consider the statistical significance of the former.

## Appendix 2

### LOST-IT hierarchy of outcomes relative to patient importance; examples included in brackets

I. Mortality

1. all cause mortality

2. disease specific mortality

II. Morbidity

1. cardiovascular major morbid events

2. other major morbid events (e.g. loss of vision, seizures, fracture, revascularization)

3. onset/recurrence/relapse/remission of cancer and other chronic diseases (e.g. COPD exacerbation, new onset of diabetes)

4. renal failure requiring dialysis

5. hospitalization, medical and surgical procedures (e.g. placement of a pacemaker, and cardioversion)

6. infections

7. dermatological/rheumatologic disorders

III. Symptoms/Quality of life/Functional status (e.g. failure to become pregnant, successful nursing/breastfeeding, depression)

Surrogate outcomes (e.g. viral load, physical activity, weight loss, cognitive function, recurrent polyps, adherence to medication)

## Supplementary Material

Additional file 1 **LOST-IT search strategy for Medline using OVID interface.** Click here for file

Additional file 2 **LOST-IT title and abstract screening form.** Click here for file

Additional file 3 **LOST-IT draft full text screening form and draft data abstraction form.** Click here for file

Additional file 4 **Details of information to be extracted about reporting of LTFU, analytical methods of dealing with loss to follow-up and LTFU statistical data.** Click here for file

Additional file 5 **Dummy tables** Click here for file
