# Supplementary material for: LOST to follow-up Information in Trials (LOST-IT): a protocol on the potential impact
Source: Trials. 2009 Jun 11;10:40. doi: 10.1186/1745-6215-10-40 (PMC2706244; doi:10.1186/1745-6215-10-40)
Supplement: Additional file 1 — LOST-IT search strategy for Medline using OVID interface. [file 1745-6215-10-40-S1.doc]

**Additional file 1:** Search strategy for Medline using OVID interface

1. randomized controlled trial.pt.

2. controlled clinical trial.pt.

3. randomized controlled trial/

4. random allocation/

5. double blind method/

6. single blind method/

7. clinical trial.pt.

8. exp clinical trial/

9. exp Clinical Trials as Topic/ or exp Randomized Controlled Trials as Topic/

10. (clin$ adj25 trial$).mp.

11. ((singl$ or doub$ or trebl$ or tripl$) adj25 (blind$ or mask$)).mp.

12. Placebos/

13. placebo$.mp.

14. random$.mp.

15. research design/

16. or/1-15

17. animals/ not humans/

18. 16 not 17

19. "2007".yr.

20. 18 and 19

21. "annals of internal medicine".jn.

22. 21 and 20

23. bmj.jn.

24. 23 and 20

25. jama.jn.

26. 25 and 20

27. lancet.jn.

28. "new england journal of medicine".jn.

29. 28 and 20

30. 27 and 20

31. 22 or 24 or 26 or 29 or 30
