# Supplementary material for: LOST to follow-up Information in Trials (LOST-IT): a protocol on the potential impact
Source: Trials. 2009 Jun 11;10:40. doi: 10.1186/1745-6215-10-40 (PMC2706244; doi:10.1186/1745-6215-10-40)
Supplement: Additional file 2 — LOST-IT title and abstract screening form. [file 1745-6215-10-40-S2.pdf]

# **LOST I**nformation in **T**rials **(LOST-IT)** study

## ***Title and Abstract screening Form*** *(version 9)*

Screener initials: ☐☐ Study ID: ☐☐☐☐ Author, year: ____________, ***2005***

Journal: ☐ AIM ☐ BMJ ☐ JAMA ☐ Lancet ☐ NEJM

1. Eligible RCT?
   - ☐ No → Exclude, *stop here*
   - ☐ Yes or unclear → Get Full Text, *answer questions below*

2. Trial described as:
   - ☐ Non-inferiority
   - ☐ Equivalence
   - ☐ Neither

3. Primary outcome clearly specified
   - ☐ Yes, one: ______________________________ *(go to q5)*
   - ☐ No, multiple primary outcomes: ______________________________ *(go to q4)*
   - ☐ None specified *(go to q4)*

4. If multiple or no primary outcome specified, select one : ______________

5. Primary outcome category # *(refer to the box)*: _______ *(e.g. II.3)*

6. The primary outcome is a:
   - ☐ Time to event outcome
   - ☐ Continuous outcome *(not time to event)*
   - ☐ Multinomial outcome
   - ☐ Binary outcome reported as rate
   - ☐ Binary outcome

7. Is it a composite endpoint?
   - ☐ Yes
   - ☐ No

8. Is it a patient important outcome?
   - ☐ Yes
   - ☐ No
   - ☐ Not clear

9. Is the result statistically significant?
   - ☐ Yes
   - ☐ No
   - ☐ Not clear

I.Mortality
1. all cause mortality
2. disease specific mortality

II.Morbidity
1. cardiovascular major morbid events
2. other major morbid events
3. recurrence/relapse/remission of cancer and other chronic diseases
4. renal failure requiring dialysis
5. hospitalizations, medical and surgical procedures
6. infections
7. dermatological/ rheumatologic disorders

III.Symptoms/Quality of life/Functional status

IV.Surrogate outcomes

*Please fill out this box for each study*

☐ **Exclude**

☐ **Get full text**

☐ **3rd reviewer needed** *(no consensus between 2 reviewers)*

If exclude, reason for exclusion:
- ☐ Not RCT
- ☐ Not eligible RCT
- ☐ Other:
