# Supplementary material for: LOST to follow-up Information in Trials (LOST-IT): a protocol on the potential impact
Source: Trials. 2009 Jun 11;10:40. doi: 10.1186/1745-6215-10-40 (PMC2706244; doi:10.1186/1745-6215-10-40)
Supplement: Additional file 3 — LOST-IT draft full text screening form and draft data abstraction form. [file 1745-6215-10-40-S3.pdf]

LOST Information in **T**rials **(LOST-IT)** study

Data abstraction Form

Screener initials:	Study ID:	Author, year: ___________________, _______
Journal: ☐ AIM ☐ BMJ ☐ JAMA ☐ Lancet ☐ NEJM		

1. Eligible RCT? ☐ No ☐ **Exclude**, *stop here*
   ☐ Yes, *type of RCT:*
   - ☐ Two arms
   - ☐ Multiple Arms
   - ☐ Factorial design
   - ☐ Report of longer follow up time
   - ☐ Report of secondary analysis
   - ☐ Report of subgroup analysis

2. Trial described as:
   ☐ Non-inferiority
   ☐ Equivalence
   ☐ Neither

3. Primary outcome clearly specified
   ☐ Yes, one: ____*(go to q5)*
   ☐ No, multiple primary outcomes: ______________________________
   ____ *(go to q4)*
   ☐ None specified *(go to q4)*

4. If multiple or no primary outcome specified, select one : __________________

5. Primary outcome category # *(refer to the guide)*: ______________________________ *(e.g. II.3)*

6. Effect on primary endpoint reported as:
   ☐ Continuous outcome exclusively ☐ **Exclude**
   ☐ Multinomial outcome exclusively ☐ **Exclude**
   ☐ Binary outcome expressed as rate exclusively ☐ **Exclude**
   ☐ Binary outcome, data not available for 2x2 table ☐ **Exclude**
   ☐ Binary outcome, data available for 2x2 table, *go to the next question*

7. Is it a composite endpoint?
   ☐ Yes, *list components*:_____________________________________
   ☐ No

8. Is it a patient important outcome?
   ☐ No ☐ **Exclude**
   ☐ Yes, *go to the next question*

9. Result statistically significant?
   ☐ No ☐ **Exclude**
   ☐ Yes

Please fill out this box for each study

☐ **Include in LOST-IT**

☐ **Exclude from LOST-IT**

☐ **3rd reviewer needed** *(no consensus between 2 reviewers)*

If exclude, reason for exclusion:
☐ Not RCT
☐ Not eligible RCT
☐ Data for the primary endpoint not available for 2x2 table
☐ Outcome not patient important
☐ Result not statistically significant

BACKGROUND INFORMATION			
10.	Number of study centers	n =	
11.	Funding<br>*Check all that Apply*	☐ Private for profit, industry only supplying medication<br>☐ Private for profit, other<br>☐ Private not for profit<br>☐ Governmental<br>☐ Not funded<br>☐ Not reported	
12.	Clinical area<br>*Check only one*	Medical<br>☐ Dermatology<br>☐ Cardiology<br>☐ Endocrinology<br>☐ Gastro Intestinal<br>☐ Hematology<br>☐ Intensive Care<br>☐ Infectious Diseases<br>☐ Neurology<br>☐ Oncology<br>☐ Psychiatric<br>☐ Renal<br>☐ Respiratory<br>☐ Rheumatology<br>☐ Other *(specify)*:	Surgical<br>☐ Cardiac surgery<br>☐ General surgery<br>☐ Obstetrics/ Gynecology<br>☐ Ophthalmology<br>☐ Orthopedic surgery<br>☐ Otorhinolaryngology (ENT: Ear Nose Throat)<br>☐ Neurosurgery<br>☐ Plastic surgery<br>☐ Thoracic surgery<br>☐ Urologic surgery<br>☐ Vascular surgery<br>☐ Other *(specify)*:
13.	Intervention<br>*Check only one*	☐ Pharmacological<br>☐ Surgery/ Invasive procedure<br>☐ Rehabilitation<br>☐ Behavioral intervention	☐ Complementary and alternative medicine<br>☐ Diagnostic test<br>☐ Other *(specify)*:
14.	Control<br>*Check only one*	☐ Standard care<br>☐ Placebo<br>☐ Pharmacological<br>☐ Surgery/ Invasive procedure<br>☐ Rehabilitation	☐ Behavioral intervention<br>☐ Complementary and alternative medicine<br>☐ Diagnostic test<br>☐ Other *(specify)*:

METHODOLOGICAL QUALITY					
15.	Concealment of Allocation<br>*Check only one*	☐ Sequentially numbered, opaque, sealed envelope<br>☐ Envelopes, other<br>☐ “Concealed”, no method described	☐ Coded medication containers<br>☐ Open random allocation schedule<br>☐ “Not concealed”	☐ Central randomization<br>☐ Quasi-randomized<br>☐ Not reported	
16.	Blinding of patients	☐ Definitely yes	☐ Probably yes	☐ Probably not	☐ Definitely not
17.	Blinding of health Care providers	☐ Definitely yes	☐ Probably yes	☐ Probably not	☐ Definitely not
18.	Blinding of data collectors	☐ Definitely yes	☐ Probably yes	☐ Probably not	☐ Definitely not
19.	Blinding of outcome adjudicators	☐ Definitely yes	☐ Probably yes	☐ Probably not	☐ Definitely not
20.	Blinding of data analysts	☐ Definitely yes	☐ Probably yes	☐ Probably not	☐ Definitely not
21.	Study stopped early for benefit	☐ Yes	☐ No		

ITT PRINCIPLE					
22.	Authors used the term ITT	☐ Yes, “ITT”	☐ Yes, “modified ITT”		☐ No
23.	Post randomization exclusion of mistakenly randomized	☐ Definitely yes	☐ Probably yes	☐ Probably not	☐ Definitely not
24.	Information about ineligibility was available at randomization	☐ Definitely yes	☐ Probably yes	☐ Probably not	☐ Definitely not
25.	Post randomization exclusions were blinded to allocation	☐ Definitely yes	☐ Probably yes	☐ Probably not	☐ Definitely not
26.	Patients for whom outcome data is available were analyzed in the arm to which they were randomized	☐ Definitely yes	☐ Probably yes	☐ Probably not	☐ Definitely not

LTFU STATEMENTS					
27.	LTFU explicitly reported	☐ Explicit statement: LTFU occurred	☐ Explicit statement: LTFU did not occur	☐ No explicit statement about LTFU	
28.	CONSORT flow diagram	☐ CONSORT diagram showing LTFU	☐ CONSORT diagram not showing LTFU	☐ No CONSORT diagram	
29.	For studies with no explicit statement about LTFU and no consort diagram	☐ Meet all "3 criteria" (see instructions)	☐ Does not meet all "3 criteria" (see instructions)	☐ N/A	
30.	LTFU reported separately for the 2 arms	☐ Yes	☐ No		
31.	LTFU reported for each time point in which effect estimate for 1ary outcome is reported in this article	☐ Yes	☐ No	☐ N/A *(only one effect estimate reported)*	
32.	Authors compared baseline characteristics of LTFU	☐ Yes, LTFU group vs. non LTFU group	☐ Yes, LTFU 1st arm vs. LTFU 2nd arm	☐ No	
33.	Implications of LTFU discussed	☐ Yes	☐ No		
34.	Method of dealing with LTFU explicitly described *(check all that apply)*	☐ Yes, survival analysis	☐ Yes, in "methods"	☐ Yes, in "results"	☐ No

35. METHOD OF DEALING WITH LTFU *(for the primary outcome)*			
	Method	Primary analysis *(check at least and only one)*	Additional analyses *(check all applicable)*
	Not applicable, no LTFU occurred	☐	☐
	Not applicable, uncertain whether LTFU occurred	☐	☐
	Unclear which method used	☐	☐
	Survival analysis (censored at the time of LTFU)	☐	☐
	Complete case analysis	☐	☐
	Worst case scenario	☐	☐
	Best case scenario	☐	☐
	None of the LFTU had the outcome	☐	☐
	All LTFU had the outcome	☐	☐
	LTFU had same outcome incidence as their group	☐	☐
	LTFU had higher outcome incidence than their group, same incidence in 2 study groups	☐	☐
	LTFU had higher outcome incidence than their group, different incidence in 2 study groups	☐	☐
	Different methods for different subgroups of LTFU:	☐	☐
	Other *(specify):*	☐	☐

LTFU STATISTICAL DATA
36. Primary outcome selected for LOST-IT (q3 or q4): ______________________________<br>☐ *Check here if outcome was reversed from positive to negative*<br>37. Follow-up time point (value): ________<br>38. Unit of time: ☐ minutes ☐ hours ☐ days ☐ weeks ☐ months ☐ years<br>39. Type of measure: ☐ fixed period ☐ mean ☐ median ☐ minimum ☐ maximum

	This table relates to the ***primary analysis*** *of the* ***primary outcome***	Intervention	Control	Total	☐ Different assumptions for different subgroups of LTFU (circle one choice, see instructions)
40.	☐ Mistakenly randomized, inappropriately excluded *(subtotal 1)*				1 2 3 4 5
41.	☐ Did not receive intervention, inappropriately excluded *(subtotal 2)*				1 2 3 4 5
42.	☐ Withdrew consent *(subtotal 3)*<br>☐ unclear whether followed up<br>☐ not followed up<br>☐ followed up, not included in the analysis *(not LTFU for LOST-IT)*				
43.	*Withdrew consent due to side effect or adverse events*				1 2 3 4 5
44.	*Withdrew consent due to other specified reason:*				1 2 3 4 5
45.	*Withdrew consent due to unclear reason*				1 2 3 4 5
46.	☐ Cross over *(subtotal 4)*<br>☐ unclear whether followed up<br>☐ not followed up<br>☐ followed up, not included in the analysis *(not LTFU for LOST-IT)*<br>☐ followed up, analyzed in a group not randomized to *(not LTFU for LOST-IT)*				
47.	*Cross over due to side effect or adverse events*				1 2 3 4 5
48.	*Cross over due to other specified reason:*				1 2 3 4 5
49.	*Cross over due to unclear reason*				1 2 3 4 5
50.	☐ Non adherent *(subtotal 5)*<br>☐ unclear whether followed up<br>☐ not followed up<br>☐ followed up, not included in the analysis *(not LTFU for LOST-IT)*<br>☐ followed up, analyzed in a group not randomized to *(not LTFU for LOST-IT)*				
51.	*Non adherent due to side effect or adverse events*				1 2 3 4 5
52.	*Non adherent due to other specified reason:*				1 2 3 4 5
53.	*Non adherent due to unclear reason*				1 2 3 4 5
54.	☐ Lost contact and no other source of outcome data *(subtotal 6)*				1 2 3 4 5

55.	☐ Other (or no reported) reason for LTFU:<br>________________________________, *(subtotal 7)*<br>☐ unclear whether followed up<br>☐ not followed up<br>☐ followed up, not included in the analysis *(not LTFU for LOST-IT)*				1 2 3 4 5
56.	☐ Different categories of LTFU reported together:<br>________________________________, *(subtotal 8)*<br>☐ unclear whether followed up<br>☐ not followed up<br>☐ followed up, not included in the analysis *(not LTFU for LOST-IT)*				1 2 3 4 5
57.	LTFU, total				

	This table relates to the *primary analysis* *of the* *primary outcome*	Intervention		Control		Total	
		Total	Events	Total	Events	Total	Events
58.	Mistakenly randomized, appropriately excluded						
59.	Did not receive intervention, appropriately excluded						
60.	Excluded as part of center exclusion						
61.	Randomized						
62.	Randomized$ (Randomized – appropriately excluded 61-(58+59+60))						
63.	Included in primary analysis						
64.	Followed-up						
65.	Measure of effect estimate	☐RR	☐HR	☐OR	☐RRR		
		☐ARR/ARI	☐Time ratio	☐Cumulative incidence ratio			
66.	Unadjusted Effect estimate; 95% CI; p value	______ (_______;_______); p=_______					
67.	Adjusted Effect estimate; 95% CI; p value	______ (_______;_______); p=_______					

68.	Inconsistency in LTFU data *(explain below)*
69.	Misunderstanding of LTFU or questionable inferences (*explain below)*
70.	Time required to complete this form: ______ min
71.	☐ Check here if we need to ask authors for numbers per study arm *(if only total numbers reported)*<br>☐ Check here if we need to ask authors for the total number of LTFU *(if not reported)*
