# Supplementary material for: LOST to follow-up Information in Trials (LOST-IT): a protocol on the potential impact
Source: Trials. 2009 Jun 11;10:40. doi: 10.1186/1745-6215-10-40 (PMC2706244; doi:10.1186/1745-6215-10-40)
Supplement: Additional file 4 — Details of information to be extracted about reporting of LTFU, analytical methods of dealing with loss to follow-up and LTFU statistical data. [file 1745-6215-10-40-S4.doc]

1. **Reporting of loss to follow-up**
   1. Whether the authors reported the occurrence of loss to follow-up. The 3 options are: explicit statement that loss to follow-up occurred, explicit statement that loss to follow-up did not occur, and no explicit statement about loss to follow-up;
   2. Whether the authors reported a flow diagram providing some indication of LTFU;
   3. Whether the authors reported loss to follow-up at each planned outcome assessment;
   4. Whether authors compared baseline characteristics of patients lost to follow up to those of the other patients;
   5. Whether the authors discussed the implications of the loss to follow-up in terms of possible weakening of the methodological quality and/or the strength of inference;
   6. Whether the authors explicitly described the method of dealing with LTFU in the methods section and in the results section.
2. **Analytical method(s) of dealing with loss to follow-up:**
3. Complete case analysis (unlike below listed methods, patients lost to follow up are excluded from the analysis and not included in the denominator);
4. Worst case scenario (assumptions: all patients lost to follow up in the treatment group had the event; none of those in the control group had it);
5. Best case scenario (assumptions: none of the patients lost to follow up in the treatment group had the event; all of those in the control group had it);
6. None of the patients lost to follow up is assumed to have had the event;
7. All patients lost to follow up are assumed to have had the event;
8. Patients lost to follow up are assumed to have had the same event incidence as their randomization group;
9. Patients lost to follow up are assumed to have had a higher event incidence than their randomization group, and the relative increase was similar in both groups (e.g., 30%); i.e., loss to follow-up is associated with the outcome but not with the intervention;
10. Patients lost to follow up are assumed to have had a higher event incidence than their randomization group, but the relative increase was different for each arm (e.g., 30% for the intervention group, 10% for the control group); i.e., loss to follow-up is; associated with the outcome and also in an independent manner with the intervention.
11. Other form of imputation;
12. Other method;
13. No analytic method used.

Elements of the intention-to-treat principle:

- Post randomization exclusions should be appropriate
- Patients should be analyzed in the arm to which they were randomized.

1. **Loss to follow up statistical data**
   1. Number randomized in each group;
   2. Number of events in each group (according to randomization);
   3. Numbers for the following subcategories at each planned outcome assessment:
      1. mistakenly randomized and inappropriately, or unclear whether appropriately, excluded;
      2. mistakenly randomized and appropriately excluded;
      3. mistakenly randomized and unclear whether excluded;
      4. withdrew consent, not followed up (due to side effects, other reasons or unclear reasons), and whether excluded from the analysis, included as no events, or included as events;
      5. withdrew consent, followed up, not included in the analysis;
      6. cross over, not followed up due to side effects, other reasons or unclear reasons), and whether excluded from the analysis, included as no events, or included as events;
      7. cross over, followed up, not included in the analysis;
      8. cross over, followed up, analyzed in the group to which they were not randomized;
      9. non adherent, not followed up (due to side effects, other reasons or unclear reasons), and whether excluded from the analysis, included as no events, or included as events;
      10. non adherent, followed up, not included in the analysis (due to side effects, other reasons or unclear reasons);
      11. non adherent, followed up, analyzed in the group to which they were not randomized (due to side effects, other reasons or unclear reasons);
      12. lost contact with patients and no other source of outcome data;
      13. “other” reason for excluding from analysis;

*PS: mistakenly randomized and appropriately excluded are not considered as loss to follow-up.*
