# Supplementary material for: LOST to follow-up Information in Trials (LOST-IT): a protocol on the potential impact
Source: Trials. 2009 Jun 11;10:40. doi: 10.1186/1745-6215-10-40 (PMC2706244; doi:10.1186/1745-6215-10-40)
Supplement: Additional file 5 — Dummy tables [file 1745-6215-10-40-S5.doc]

**Additional file 5:** Dummy tables

**Table 1:** Dummy table for the percentage of RCTs with the assumption that the event incidence among LTFU participants (relative to observed participants) increased and the relative increase (RI_LTFU/FU_) is higher in the intervention group

|  |  | RI_LTFU/FU (control)_ | | |
| --- | --- | --- | --- | --- |
|  |  | 1 | 1.5 | 2 |
| RI_LTFU/FU (intervention)_ | 1.5 |  | ------ | ------ |
|  | 2 |  |  | ------ |
|  | 3 |  |  |  |

**Table 2:** Dummy table for the mean change in effect size across RCTs with the assumption that the event incidence among LTFU participants (relative to observed participants) increased and the relative increase (RI_LTFU/FU_) is higher in the intervention group

|  |  | RI_LTFU/FU (control)_ | | |
| --- | --- | --- | --- | --- |
|  |  | 1 | 1.5 | 2 |
| RI_LTFU/FU (intervention)_ | 1.5 |  | ------ | ------ |
|  | 2 |  |  | ------ |
|  | 3 |  |  |  |

**Table 3:** Dummy table for the percentage of RCTs losing statistical significance with the assumption that event incidence among LTFU participants (relative to observed participants) increased in the intervention group and decreased in the control group

|  |  | RD_LTFU/FU (control)_ | | |
| --- | --- | --- | --- | --- |
|  |  | 0.66 | 0.5 | 0.33 |
| RI_LTFU/FU (intervention)_ | 1.5 |  |  |  |
|  | 2 |  |  |  |
|  | 3 |  |  |  |

**Table 4:** Dummy table for the mean change in effect size across RCTs with the assumption that event incidence among LTFU participants (relative to observed participants) increased in the intervention group and decreased in the control group.

|  |  | RD_LTFU/FU (control)_ | | |
| --- | --- | --- | --- | --- |
|  |  | 0.66 | 0.5 | 0.33 |
| RI_LTFU/FU (intervention)_ | 1.5 |  |  |  |
|  | 2 |  |  |  |
|  | 3 |  |  |  |
